# Penetrating Barriers: Noncontact Measurement of Vital Bio Signs Using Radio Frequency Technology

**DOI:** 10.3390/s24175784

**Published:** 2024-09-05

**Authors:** Kobi Aflalo, Zeev Zalevsky

**Affiliations:** 1 Faculty of Engineering, Bar-Ilan University, Ramat Gan 5290002, Israel; 2 ELTA Systems, Israeli Air Industries, 100 Yitzchak Hanasi Blvd, Ashdod 7762403, Israel

**Keywords:** microwave, noninvasive, respiration rate, heart rate

## Abstract

The noninvasive measurement and sensing of vital bio signs, such as respiration and cardiopulmonary parameters, has become an essential part of the evaluation of a patient’s physiological condition. The demand for new technologies that facilitate remote and noninvasive techniques for such measurements continues to grow. While previous research has made strides in the continuous monitoring of vital bio signs using lasers, this paper introduces a novel technique for remote noncontact measurements based on radio frequencies. Unlike laser-based methods, this innovative approach offers the advantage of penetrating through walls and tissues, enabling the measurement of respiration and heart rate. Our method, diverging from traditional radar systems, introduces a unique sensing concept that enables the detection of micro-movements in all directions, including those parallel to the antenna surface. The main goal of this work is to present a novel, simple, and cost-effective measurement tool capable of indicating changes in a subject’s condition. By leveraging the unique properties of radio frequencies, this technique allows for the noninvasive monitoring of vital bio signs without the need for physical contact or invasive procedures. Moreover, the ability to penetrate barriers such as walls and tissues opens new possibilities for remote monitoring in various settings, including home healthcare, hospital environments, and even search and rescue operations. In order to validate the effectiveness of this technique, a series of experiments were conducted using a prototype device. The results demonstrated the feasibility of accurately measuring respiration patterns and heart rate remotely, showcasing the potential for real-time monitoring of a patient’s physiological parameters. Furthermore, the simplicity and low-cost nature of the proposed measurement tool make it accessible to a wide range of users, including healthcare professionals, caregivers, and individuals seeking to monitor their own health.

## 1. Introduction

A variety of noninvasive detection technologies for vital bio signs have been proposed in the literature, some of which require direct contact, which can be difficult in some cases; these technologies include electrocardiograms (ECG) [1,2,3] and wearable optical fibers [4]. There are also techniques to monitor health by placing patches directly on the skin. In a recent study by Yoon et al. [5], a novel multifunctional hybrid skin patch was introduced. This patch is designed for the electrochemical analysis of sweat glucose levels while simultaneously monitoring ECG. Khan et al. [6] explored advanced techniques for developing flexible and wearable sensing electronics in healthcare, emphasizing printing technologies that allow for cost-effective manufacturing of flexible substrates. They highlight the use of nanoscale materials that enhance sensitivity in detecting biomarkers in biological fluids such as sweat. These biocompatible sensors enable real-time data transmission through wireless communication, facilitating remote health monitoring. A study by Matko and Milanović [7] presents a novel temperature-compensated capacitance–frequency converter that achieves high-resolution capacitance measurements. This innovative method employs a single quartz crystal within a switching oscillator circuit, significantly reducing the temperature influence on frequency changes across a range of 0 to 50 °C. The authors highlight the potential of this converter to achieve Zepto-Farad resolution, making it particularly promising for applications in various fields, including biosensor technology and the noninvasive monitoring of vital signs. By addressing the challenges of temperature compensation and measurement accuracy, this work contributes to the development of more reliable and sensitive noninvasive devices for physiological monitoring.

Piezoelectric nanogenerators (PENGs) are gaining attention due to their ability to continuously monitor vital signs by converting the mechanical energy from physiological movements into electrical energy. Utilizing the principle of piezoelectricity, PENGs can harvest energy from actions such as heartbeat and respiration [8], enabling integration into wearable devices such as smartwatches for real-time health monitoring. Additionally, PENGs can power biosensors without the need for external batteries, offering a self-sustaining solution for uninterrupted health tracking. Their lightweight and compact design further enhances their suitability for wearable applications, marking a significant advancement in health technology and energy harvesting. Piezoelectric materials, especially those based on cellulose nanocrystals (CNCs), are increasingly utilized for the noninvasive monitoring of vital signs such as respiration and heart rate. An all-3D-printed pyro-piezoelectric nanogenerator (Py-PNG) can harvest energy from both mechanical and thermal sources [9], facilitating its use in wearable devices for real-time health monitoring. The biocompatibility and biodegradability of CNCs enhance their suitability for continuous monitoring in healthcare applications, providing valuable insights into an individual’s health status without requiring external power sources. In this study, we use a simple piezoelectric sensor as a reference to verify the performance of our noninvasive, contactless measurements. While there are other sensors that do not require contact, they come with some limitations in terms of penetrating through tissues, such as infrared cameras [10] and lasers [11,12].In prior noninvasive optical sensor research, the use of laser technology to monitor vital biological signals via secondary speckle patterns was explored [11]. This method involved directing a laser beam onto two specific areas of a person’s body: the heart and the wrist. Two synchronized cameras were then used to capture the resulting speckle patterns. By analyzing the differences between the peaks, their distances, and their physical separation on the human body, it was possible to calculate the pulse wave velocity (PWV) [13]. However, this approach has certain limitations, including the inability to transmit through tissues and barriers, which could potentially provide additional critical data.

In certain situations, it is crucial to obtain essential details about the internal organs. Ventricular tachycardia (VT) is a life-threatening arrhythmia (irregular heartbeat). A novel method is presented in [14] to automatically extract the key information from pace-mapping data with the automated detection of paced heartbeats from the surface ECG signals by using wavelet detection on pacing spikes and combined time/energy criteria. A paced-ECG detector was developed to detect VT. The employment of a noninvasive sensor capable of penetrating tissues could yield a signal akin to an ECG, which could subsequently be analyzed. When the patient is positioned behind an opaque obstacle, the laser beam will not have any interaction with the patient’s tissues. Here, a different form of radiation, such as radio frequencies (RFs), is needed. In the context of radar systems [15], the Doppler effect manifests as a frequency shift in the reflected signal when it encounters a moving target. This shift is directly proportional to the velocity of the target and can provide valuable information about its movement. Microwave radiation is proposed for remote sensing behind walls [16]. Microwave signals can safely penetrate clothing and reveal concealed threats, e.g., explosives and firearms, without imposing any health risks or side effects. An additional study showcases the utilization of RF speckle-based remote sensing using pulse-Doppler radar for the purpose of vibration detection in a stationary target [17]. The outcomes have been validated through the successful differentiation of various engine states. This marked the initial advancement toward the real-time identification of human vital signs through RF speckle patterns. An X-band microwave life-detection system was developed [18] and found to be able to detect respiration and heart rate using cinder blocks for patients lying on the ground at a distance of about 30 m. The advantages of noncontact vital sign detection using a microwave Doppler radar signal of 2.5 GHz at a distance of 1 m have been demonstrated [19]. Another study uses a continuous-wave radar system for a life detection system using an automatic clutter cancellation mechanism [20]. Recent studies have investigated the use of compact continuous-wave (CW) radars for the detection of small movements. These radars leverage Doppler displacement for the detection of respiration rates [21] at 2.4 GHz and small target displacements [22] using IQ (in-phase and quadrature) signal demodulation. Techniques using CW RF radiation have demonstrated promising results, suggesting the potential for utilizing this technology in the detection of abdominal movements.

In the realm of RF techniques for remote sensing, a recent study investigated the use of cyclostationary signal processing for vital sign detection [23]. Here, the reflected signal was highly affected by random body motion. The reflected signal was corrupted by noise and random body movements, and traditional Fourier analysis failed to identify the heart and respiration frequencies. The experimental results revealed that the cyclostationary detection performance is dependent on the target distance and signal-to-noise ratio (SNR) values. A demonstration of frequency-modulated continuous wave (FMCW) radar for the noncontact vital sign monitoring of multiple individuals is shown in [24]. This technology has been found to be particularly effective in tracking vital signs such as respiration and heartbeat, even in environments filled with multiple objects and noise. The study further highlights the radar’s capability to accurately localize humans in a noisy environment, including both static and vibrating objects. There are several techniques for the remote sensing of vital signs using RF, each with its own limitations and unique approach. In [25], the remote sensing of vital signs is discussed, and two key methods are radar-based and WiFi-based monitoring. Radar systems, including Doppler, ultra-wideband (UWB), and frequency-modulated continuous wave (FMCW) radars, measure chest movements through electromagnetic echoes, operating in the microwave frequency range. While these systems provide high precision, their high costs limit accessibility. In contrast, WiFi-based monitoring utilizes channel state information (CSI) from multiple subcarriers to detect respiratory movements, leveraging existing WiFi infrastructure for a more cost-effective solution. However, this method faces challenges in terms of accuracy due to environmental factors and the influence of user location and body orientation on detection reliability. In [26], the authors utilized a combination of singular-spectrum analysis (SSA) and variational mode decomposition (VMD) to enhance the remote sensing of vital signs, such as respiration and heartbeat, using ultra-wideband (UWB) radar technology. SSA helps eliminate noise peaks around the heartbeat rate, while VMD allows for the adaptive decomposition of radar echo signals, enabling the high-accuracy extraction of vital sign information. However, limitations include challenges from weak heartbeat amplitudes and environmental noise, which can impact measurement accuracy. The UWB radar operates effectively within a frequency range that supports strong penetration and high resolution for noncontact monitoring. Another study [27] explored remote sensing methods for vital signs, primarily using radar and WiFi technologies. FMCW radar at 77 GHz extracts vital parameters through signal processing, achieving high accuracy in respiratory rate estimation. WiFi-based solutions utilize CSI and machine learning for monitoring. However, these methods have limitations, including sensitivity to environmental conditions and the need for stationary subjects. Radar techniques are effective only when subjects are idle, while WiFi systems may require higher power consumption due to using separate transmitting and receiving devices. Despite these challenges, these technologies offer promising avenues for contact-free health monitoring. In [28], the authors introduce a method for the remote sensing of vital bio signs, specifically respiration, using CSI from commercial WiFi signals at a frequency of 5.8 GHz. The BreatheBand system operates with a sampling rate of 200 Hz, allowing for noncontact monitoring without wearable devices. While this approach offers advantages over traditional methods, such as reduced intrusiveness, it faces limitations, such as potential inaccuracies in non-line-of-sight scenarios and reliance on existing WiFi infrastructure. Overall, BreatheBand represents a significant advancement in unobtrusive health monitoring. The authors of [29] explored RFID technology for the remote sensing of vital bio signs, particularly for non-intrusive respiration monitoring. RFID systems use passive tags that reflect radio frequency signals, enabling the detection of subtle chest movements. The advantages include lightweight design, cost-effectiveness, and multi-user support via unique tag IDs. However, limitations arise from multi-path effects in dynamic environments, where reflections from nearby movements can introduce noise, complicating signal interpretation. By operating typically in the UHF frequency range (860–960 MHz), RFID systems are effective for short-range applications but may face interference from other RF sources. Addressing these challenges is essential for enhancing the reliability of RFID-based bio sign monitoring. The authors of [30] discussed a frequency comb continuous wave (FCCW) bio radar system for the remote sensing of vital signs, specifically detecting the respiration of individuals trapped under debris. Operating at a center frequency of 1.3 GHz, this method transmits evenly spaced frequency components, enabling the high-resolution detection of small breathing motions. However, its 60 MHz bandwidth limits range resolution to 2.5 m, potentially missing finer respiratory movements. Additionally, the reliance on the Doppler effect may present challenges in noisy environments or with interference from other sources.

When comparing laser-based sensing to RF-based sensing, it is crucial to recognize that these two methodologies are fundamentally distinct, not only in terms of signal generation but also with respect to their underlying sensing mechanisms. Both techniques utilize two instruments: one to illuminate the target and the other to capture the effect of this illumination. In an optical system, this involves a beam former (laser) and a camera, whereas in an RF system, there are transmitting and receiving antennas, which can be identical. In contrast, in an optical system, we cannot interchange a laser with a receiving antenna due to their different structures and operations. Although both operate on the principle of transmitting electromagnetic waves at different wavelengths (nanometers for optical systems and centimeters for microwaves), their effects on materials differ. Lasers cannot penetrate opaque materials, whereas RF can. Lasers mostly reflect off of smooth surfaces (relative to their wavelength), whereas RF is primarily reflected by conducting surfaces. The use of a laser beam involves a direct, coherent beam of narrow spectral width (monochromatic), whereas RF can utilize a wider spectrum and easily modulate the signal. When using a laser, we can measure the reflected pattern, also known as the speckle pattern [12]. In the case of RF, we can measure a single phase shift in the reflected beam and capture limited information on the target’s behavior at the beam’s intersection. The captured data from an optical system come in the form of a spatial matrix over a time period, whereas in RF, the captured data measure the RF field as a single numerical value over a time period.

In this study, we propose the utilization of a CW radar operating at a frequency of 2.4 GHz. Microwave radiation, endowed with the capability to easily penetrate walls and tissues while experiencing minimal attenuation, exhibits a range extension of up to 10 m from the subject. This attribute equips the system to detect concealed moving targets remotely by identifying distinctive Doppler shifts that are separate from the transmitted signal. The scrutiny of frequency shift and amplitude fluctuations facilitates the identification of both motion and its velocity. Considering that abdominal movements typically manifest at relatively low frequencies, this technique is adept at scrutinizing vital signs within the human body.

One of the key advantages of using microwave radiation in the 2.4 GHz range to detect vital bio signs, as opposed to other types of radiation, is its pervasive presence in today’s world. Despite its common use, our proposed method has proven effective in detecting vital bio signs in both controlled (such as an anechoic chamber) and uncontrolled environments. For safety, we use a very low level of power, thus significantly reducing any potential harm to humans. It is also noteworthy that, unlike other methods requiring a direct line-of-sight from the sensing device to the subject without any obstacles, our method enables the detection of subjects even behind opaque obstacles. This makes it a viable solution for search and rescue operations.

Our proposed method, unlike the previously reviewed research, is a distinct sensing technique that enables near-field detection without minimum range limitations. It can sense all micro-movements, including those perpendicular to the radiation direction, but it cannot determine the range and direction of targets—only that they are within the beam’s coverage. Unlike typical radar systems, which use multiple radiating elements, our design employs a single antenna for transmission and another for reception, minimizing multi-path interference due to negligible side lobes. We utilize continuous radiation originating from a single power source with a synchronized phase at a low-power frequency, simplifying the spectrum and allowing for easy modification and amplification to overcome interference. Our narrow spectral range results in low bandwidth and minimal white noise, making our method more efficient compared to conventional radar systems that operate over a wider spectral range.

Our work encompasses the hardware development of this CW radar, which is deployed for various scenarios involving minor physical motions. We compare the results obtained from measurements and the processing of the outcome of experimental trials. Additionally, we delve into the exploration of diverse algorithms capable of detecting and extracting highly saturated, low-amplitude signals amid background noise. Our study encompasses the technique of wavelet-based analysis against the more conventional approach of Fourier-based spectrogram utilization for the examination of nonstationary low-frequency signals across temporal dimensions.

## 2. Methods

This preliminary preparation, coupled with a theoretical framework drawn from prior research, was used to assess the feasibility of the proposed methodology. It also introduces various analytical tools that can facilitate the interpretation of results and contribute to a more comprehensive understanding of the subject matter.

The simulations in this study are designed to predict the expected behavior of the desired signals, thereby facilitating the preliminary testing of the algorithms used. In order to validate the results of these simulations, a series of experiments was conducted based on various scenarios involving measurements of breathing and heart rate over time. Our proposed method differs from the typical radar-based approach. Unlike radar, we classify this method as a distinct sensing technique. It enables detection in the near-field region without minimum range limitations. We can sense all micro-movements, including those perpendicular to the direction of radiation. However, unlike typical radar systems, we cannot determine the range and direction of the target; we can only ascertain that the target is within the beam’s coverage. Our detection capability is limited to any nonstationary targets inside the beam. Typical radar systems are composed of multiple radiating elements (phased array) that do not require calibration. In contrast, our antenna design features negligible side lobe levels, resulting in almost no effect from multi-path interference. In our method, we use only one antenna for transmission and another for reception. Our method utilizes continuous radiation in a single continuous wave rather than pulses, simplifying the spectrum to a single low-power frequency that can be easily modified and amplified to overcome interference. Additionally, since we use only a narrow spectral range, our bandwidth is very low, resulting in minimal white noise $k_{B}TBF$, where $k_{B}$ is the Boltzmann constant, *T* is the ambient temperature, and *B* is our system bandwidth in Hz. This is important because our spectrum is narrower compared to conventional radar systems that modulate their radiation and use a wider spectral range.

Ethics approval for the study was provided by the institutional review board of Bar-Ilan University. All participants provided informed consent for participation in the study. The experiments were carried out in accordance with relevant guidelines and regulations. This study involved exposure to low-power, non-ionizing radiation that was expected to last for about 1 min. The participants’ informed consent to publish the image in an online open access publication was obtained.

It is important to note that the use of microwave-sensing technologies for monitoring physiological signals presents unique privacy challenges. Unlike laser-based systems, which require a direct line of sight and can be easily shielded, microwave signals can penetrate walls and other barriers. This capability, while advantageous for remote monitoring, raises significant concerns regarding the potential for unauthorized surveillance and the collection of sensitive health information without the subject’s knowledge. In order to address these privacy concerns, we emphasize the importance of transparency in communicating the capabilities and limitations of microwave-sensing technologies to any potential subjects and the broader community. Researchers must ensure that individuals are informed about the potential for detection, even when they are not actively participating in the study. Furthermore, adherence to existing privacy laws and regulations is essential to protect individuals’ rights.

By integrating these considerations into our methodology, we aimed to foster a responsible approach to the application of microwave-sensing technologies in health monitoring, ensuring that the benefits of such innovations do not come at the expense of individual privacy rights.

The following sections will discuss the simulations and experimental validation. Our algorithms and the logic of the signal processing are as follows: We captured analog measurements of voltage levels at a 32-bit resolution, along with their corresponding time in milliseconds, as the data. The sampling rate achieved using our equipment is approximately 12 kHz, which is sufficient for vital bio sign detection. According to the well-known Nyquist–Shannon sampling theorem [31], the signal bandwidth should not exceed half of the sampling frequency, thereby limiting our spectrograms and scalograms to one-half of the sampling frequency. We developed a program in MATLAB version R2023a (MathWorks, Natick, MA, USA), accompanied by additional useful functions, to process the data and extract a continuous wavelet transform (CWT) scalogram decomposition. As shown later in Section 2.2, we utilized the “Daubechies-3” wavelet in general and “Symlet” wavelets for the detection of ECG signals. Most of our extracted data were obtained by observing the scalogram cross-section through the frequency and time axes, where we investigated the points of interest that indicate high magnitudes, signifying detected movements.

### 2.1. Microwave Spectra of Tissues

In the following section, we investigate the theoretical properties of human tissue. Our goal is to evaluate the potential use of 2.4 GHz, a frequency in the microwave radiation range, due to its ability to penetrate through tissue with minimal losses. We aim to present a straightforward and fundamental analysis of the interaction between tissue and microwave radiation. The complex dielectric properties of tissues were collected for investigation. Spectral tissue data were measured and collected from the literature [32], covering a range of 25 to 8500 MHz at 37 °C for skin, muscle, and fat. Another more recent study [33] examined 54 types of tissue and showed their properties to be in the range of 500 MHz to 20 GHz. The latter study [33] investigated analytical models of dielectric properties in the range of 500 MHz to 20 GHz and showed good agreement with the previous measurements in [32] using a multi-pole Debye model fit. Table 1 presents the calculated tissue parameters that were fitted using the Debye model. The complex dielectric constant ε includes the relative loss factor $K^{\prime \prime }$, where we wish that the effect of losses for 2.4 GHz will be minimal. The complex permittivity ε is expressed in the form of Equation (1):

(1) $$\varepsilon \;=\varepsilon^{\prime }-i\varepsilon^{\prime \prime }\;,$$

where $\varepsilon^{\prime }$ is the real part of the relative dielectric constant and $\varepsilon^{\prime \prime }$ is its imaginary part. The loss factor represents the loss of electric field energy due to frictional losses by charge flow and dipolar rotation. The ratio *K* of the actual permittivity of the material to that of a vacuum is shown in Equation (2):

(2) $$K\;=K^{\prime }-iK^{\prime \prime }=\varepsilon /\varepsilon_{0}\;,$$

The loss factor $K^{\prime \prime }$ can be expressed by Equation (3), where ρ is the resistivity in units of Ω/m, ω is the angular frequency in radians per second, and σ is the conductivity in 1/Ωm.

(3) $$K^{\prime \prime }=1/\rho \omega \varepsilon_{0}\;=\sigma /\omega \varepsilon_{0},$$

**Figure 1:**
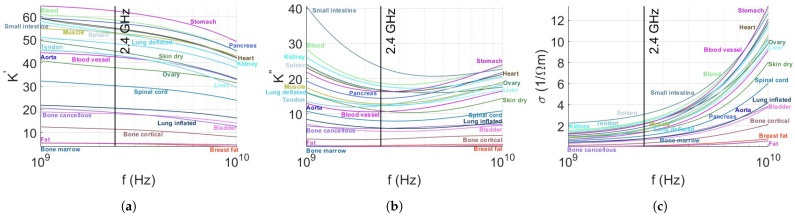
The variation in the complex dielectric constant: real (**a**), imaginary (**b**), and conductivity (**c**) with microwave frequency, with emphasis on the 2.4 GHz frequency indicated by a black vertical line, for 23 different tissues located in the abdominal and upper chest regions.

The dielectric loss factor is a measure of the energy absorbed in the medium as an electromagnetic wave passes through that medium. In the ideal case, the dielectric loss factor is zero. A low value of the dielectric loss factor is ideal for applications that require penetration through tissues. The values extracted from the tabulated data in [33] were calculated at neighboring frequencies and are depicted in Figure 1. The conductivity shown in Figure 1c is low, suggesting that most of the radiation is propagating and only a small part of it is lost or absorbed in the medium of propagation. Figure 1a displays the dielectric constant for each tissue. Figure 1b illustrates the behavior of $\varepsilon^{\prime \prime }$, which is directly proportional to the dielectric loss factor $K^{\prime \prime }$. The loss factor is predominantly at its lowest value in the region of 2.4 GHz, indicating a low loss factor that suggests minimal energy losses.

In our experiments, the test subject wore a cotton t-shirt, which may have influenced the results due to its dielectric properties. The literature on the microwave dielectric properties of textiles [34,35] indicates that for pure cotton fabric at a radiating microwave frequency of approximately 2.4 GHz under ambient conditions of 21 ± 0.2 °C and relative humidity of 80 ± 2.5%, the real part of the dielectric properties is 1.46, with no imaginary part and no loss tangent. This suggests that the cotton material is nonconductive and exhibits negligible losses. Regarding the color of clothes, we acknowledge that different dyes may have varying chemical compositions, which can affect moisture absorption and, consequently, the dielectric properties of the fabric. In our case, we lack such information about the clothing of the test subject, so we assume nonconductive properties without losses.

### 2.2. Analysis of Nonstationary Signals

In order to analyze the signals that we captured, we utilized a few techniques that are used to decompose the signal and extract key components from it, which assisted us in evaluating the subject’s condition. The measured signal is saturated with random noise and highly affected by other body movements. Since the analyzed signal is nonstationary, there are two methods for analyzing such a signal: short-time Fourier transfer (STFT) [36] and contentious wavelet transfer (CWT) [37]. Wavelets are a relatively recent development in applied mathematics; the scalogram with wavelet decompositions works by correlating a window function (wavelet) with the nonstationary temporal signal, where there is good frequency resolution in the lower frequencies with poor time resolution and good time resolution in higher frequencies with poor frequency resolution at higher frequencies; this is highly dependent on the wavelet function selection. Ideally, the wavelet choice should match the desired signal temporal shape. This wavelet property is suitable for slowly varying signals over time, such as body movements. According to [38], an instance of suggested wavelets suitable for analyzing ECG signals are “Daubechies-3” and “Symlets-3” wavelets, which were found to perform best when analyzing ECG signals. In [39], the simulation results obtained using a “Symlet” wavelet were shown to achieve an accuracy of 87.5% in recognizing electrocardiogram signals.

The efficacy of wavelet denoising [40] hinges upon the availability of prior information or the established properties of a signal. This knowledge is encapsulated within an operator designed to attenuate noise while retaining the integrity of the underlying signal. Ideally, the joint probability distribution of both the signal and noise is presumed to be known, thereby facilitating a more precise denoising process.

## 3. Simulation

**Figure 2:**
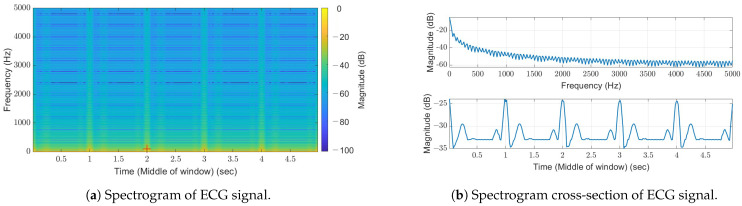
This method, as evidenced by the spectrogram (**a**) and its cross-section (**b**) at the point of interest, is not optimal for discerning details about low frequencies due to the lower resolution at these frequencies. Since the spectrogram is based on a Fourier transformation of a finite signal within a window function, it has several frequency components that are clearly visible in the horizontal blue lines (**a**).

Prior to the experimental part, we planned to perform preliminary simulations, which are discussed in the following section. These simulations helped us validate our algorithm and demonstrate its outcome under ideal conditions. In the initial phase, we created a synthetic ECG signal based on the literature [3] that mimics a normal heartbeat at a frequency of 60 beats per minute. This signal, known as the QRS complex, includes the P wave (the first wave seen on the ECG tracing), the Q wave (the first negative deflection after the P wave), the R wave (the first positive deflection following the Q wave), and the S wave (the negative deflection following the R wave). We used this ECG signal to validate our hypothesis that the conventional short-time Fourier transformation (STFT) method is less effective for low-frequency signals. This is due to the lower resolution it provides for these signals, which are commonly associated with human abdominal movements. An example of STFT decomposition in a spectrogram is shown in Figure 2a. Here, the heartbeats are visible in the higher frequencies, but they are almost unnoticeable in the lower frequencies. Figure 2b is the cross-section of Figure 2a located at the red cross in the middle; this reveals that the majority of the high-magnitude frequency components are found at the lowest frequencies. However, the resolution at these frequencies is low, making this method unsuitable for monitoring bio signs.

**Figure 3:**
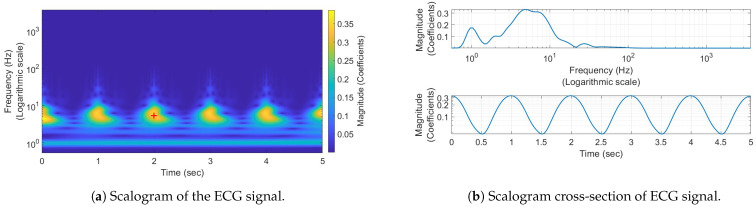
The scalogram (**a**), which illustrates the time-dependent frequency components of the ECG signal’s pulses, features a red cross as a noteworthy point that is examined in the cross-section (**b**), showing all axes information at the cross position.

An alternative method for detecting signals from the human body, which are typically in the low-frequency range of approximately 1 to 3 Hz, involves the use of continuous wavelet transformation (CWT). This decomposition, as depicted in a scalogram in Figure 3a, is more suitable for low-frequency applications due to its ability to achieve a high resolution in such scenarios. The frequency axis in this figure is on a logarithmic scale, making all the harmonics of the ECG clearly visible and distinguishable. Figure 3b, which is a cross-section of Figure 3a at the red cross located in the middle, shows that all the frequency components are distinct and noticeable. This characteristic makes this method well-suited for monitoring vital bio signs.

## 4. Experimental Validation

In this section, we present the experimental validation of our proposed system. The prototype, while currently relatively expensive, has the potential for significant cost reduction upon mass production. Our system stands out from conventional sensors due to its ability to detect vital bio signs through opaque obstacles without requiring direct contact or line-of-sight. Furthermore, it is unique among radar-based techniques that utilize microwave radiation due to its capability to detect movements in all directions. These advantages underscore the potential of our system in practical applications, particularly in scenarios where the noncontact and omnidirectional detection of vital bio signs is required. The following subsections detail the experimental setup, data collection process, and an analysis of the results, demonstrating the effectiveness and potential of our proposed method.

**Figure 4:**
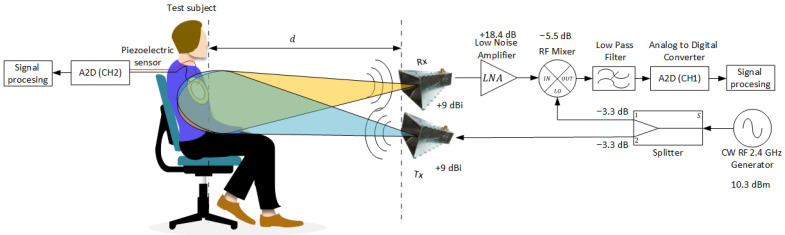
Depiction of the RF trajectory and the subject’s position, with both antennas aimed at the same point. The test subject is equipped with a piezoelectric sensor on the torso, providing an extra reference for chest movements.

In order to authenticate our study, we have constructed a prototype of a remote sensing system, as outlined above. A typical radar system is composed of several key components: a transmitter, a receiver, and signal-processing hardware or software. The transmitter’s role is to generate a continuous waveform, which is then focused toward the target by a directional antenna. Once the signal is received, it is converted from the transmission frequency to an intermediate frequency or baseband by the receiver. The signal processing component is crucial for filtering out clutter and out-of-band noise, allowing the desired signal to pass through and enabling the extraction of information from the signal. The remote sensing system, as depicted in Figure 4, features two transmitting and receiving antennas that operate concurrently. These antennas are synchronized to the same phase because they both originate from a single RF generator. This simultaneous operation and synchronization ensure the effective functioning of the radar system.

The current setup employs a Keysight model E8257D signal generator (Keysight Technologies, Santa Rosa, CA, USA) as the RF source, which is connected to a broadband, linearly polarized LB-20180 horn antenna (A-Info, Irvine, CA, USA) via an RF chain. The RF chain comprises a connectorized RF mixer (Mini-Circuits, Brooklyn, NY, USA, p.n ZEM-4300+), a low-noise amplifier (Mini-Circuits, Brooklyn, NY, USA, p.n ZX60-123LN-S+), a splitter (Mini-Circuits, Brooklyn, NY, USA, p.n ZB2PD-63-S+), and a low-pass filter with a cutoff frequency approximately twice the desired maximum sampling rate of 12 kHz. The conversion from analog to digital (A2D) is executed by a high-speed Arduino DUE board, capable of sampling analog signals at a rate of 12 kHz. The resulting digital signal is transferred to a computer in real time via a universal serial bus (USB) interface. The digital data are then analyzed using MATLAB software version R2023a (MathWorks, Natick, MA, USA), where wavelet decomposition algorithms and preliminary preprocessing techniques are employed to extract key information.

### 4.1. Guidelines for RF Parameter Determination

For the optimal selection of RF parameters, we account for the loss and amplification at each step in the RF chain to determine the appropriate parameters to be used. Given that the RF cables in use are short, their attenuation is negligible. The considerations are as follows: when considering the transmission chain, for minimal conversion losses of the mixer in use (p.n ZEM-4300+), the signal generator must transmit a power of at least 10.3 dBm. This power is then almost halved (−3.3 dB) by the splitter. One end of the splitter goes to the transmitting antenna, while the other goes to the mixer’s intermediate frequency (IF) input. The IF input of the mixer is then 7 dBm, which is the required level for the proper function of this mixer at the frequency *f* that we chose. Due to the antenna gain of 9 dBi, the output transmission power is about 16 dBm. The free-space path loss (FSPL) for a distance of 2d backward and forward from hitting the subject and reflecting backward is calculated using Equation (4), which gives a loss of 28 dB for a distance of 1 m between the antennas and the target. The gain of both the transmitting $G_{t}$ and receiving $G_{r}$ antennas are identical.

(4) $$FSPL\;\left(\mathrm{dB}\right)=20\log_{10}\left(2d\right)+20\log_{10}\left(f\right)+20\log_{10}\left(\frac{4\pi }{c}\right)-G_{t}-G_{r},$$

Since the level of reflection from the subject can be different from one subject to another, we define the received power level in the reception chain as an unknown parameter *x*. In the reception chain, the low-noise amplifier (LNA) amplifies the signal by 18.4 dB, which then goes to the RF mixer, which has a conversion loss of −5.5 dB. The signal that reaches the low-pass filter (LPF) is at the level of *x* minus 12.84 dB, where *x* is the received input power. The noise level of the reception chain can be estimated by evaluating the thermal noise level $k_{B}TBF$, where $k_{B}$ is the Boltzmann constant, *T* is the ambient temperature in Kelvin units, *B* is our system bandwidth in Hz (which is 6 kHz), and *F* is the amplifier noise figure at its first stage, with a value of 2.4 dB. The noise level in our system at an ambient temperature of 27 °C is −133.6 dBm; the received signal cannot be detected below this level. For a rough estimation of our capabilities of detection at different distances, *d*, in the far field, we utilized the well-established Friis formula [41] (Equation (5)) to calculate the reflected received power $P_{r}dBm$; this is relevant for areas where the wavelength is significantly greater than the distance λ≫d. In our specific case, when the wavelength is 12.5 cm greater than that distance, the subject is considered to be in the far-field region.

(5) $$P_{r}\;\left(\mathrm{dBm}\right)=P_{t}\left(\mathrm{dBm}\right)+G_{t}+G_{r}+20\log_{10}\left(\frac{\lambda }{4\pi d}\right),$$

**Figure 5:**
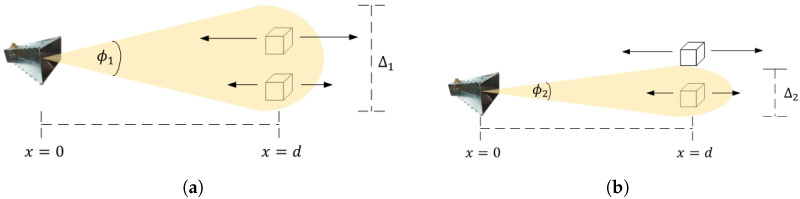
The separation of targets in close proximity is greatly influenced by the beam width and the targets’ distance from the antenna. Scenario (**a**) demonstrates a situation where the target of greater magnitude obscures the slower, less intense target. On the other hand, scenario (**b**) depicts a case where the targets are distinct and separated. In this scenario, since the beam is narrow and aimed toward the slower target, it can be detected.

The ability to detect movement at a distance, *d*, is greatly influenced by the beam width of the antenna and any nearby movements that could interfere with the detection. If these movements produce a larger magnitude, they can overshadow smaller ones, as illustrated in Figure 5a. Therefore, for our system to function effectively, it is advisable to use a narrow beam that focuses solely on the area of interest, as shown in Figure 5b. With a specified beam width, we can calculate the separation distance, Δ, of two targets located at a distance of *d*.

(6) $$\Delta_{n}=d\cdot \arctan\left(\phi_{n}\right),$$

**Figure 6:**
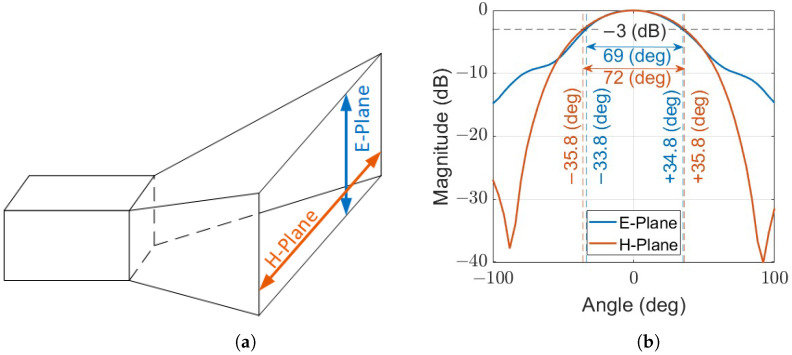
Illustration of the linearly polarized antenna that was used, with its corresponding horizontal (H) and vertical (E) planes (**a**) and the corresponding beam pattern in each plane (**b**). (**a**) Illustration of the rectangular aperture linearly polarized antenna and its corresponding linear planes. (**b**) Beam pattern of the deployed antenna across both horizontal (H) and vertical (E) planes.

The antenna utilized was a linearly polarized horn antenna. It operates at a working frequency of 2.4 GHz and exhibits a voltage standing wave ratio (VSWR) of 1.54. The antenna’s E and H planes are illustrated in Figure 6a. It also features a beam width at the 3 dB level of 72 degrees for the H-plane and 69 degrees for the E-plane. These characteristics are depicted in Figure 6b. We utilized this information to determine the optimal placement of the subject or the antenna for enhanced target separation.

In order to calculate the furthest distance we can detect for a given SNR, we reformulate the Friis formula Equation (7). This new form allows us to estimate the maximum distance based on the SNR. The level of SNR(x=d) represents the SNR measured at position x=d, while $SNR(x=d_{max})$ denotes the SNR at the greatest possible distance for detection, which is zero. This results in an equation for estimating $d_{max}$, the maximum detectable distance.

(7) $$d_{max}=d\sqrt[{4}]{\left(\frac{\left(10^{SNR(x=d)/10}\right)}{\left(10^{SNR(x=d_{max})/10}\right)}\right)},$$

The calculation of $d_{max}$ is as follows: first, we estimate our system’s frequency response for a given input power, $P_{in}$, as described later in Section 4.4. Then, we find the SNR at a given distance, *d*, denoted as SNR(x=d). By combining these elements in Equation (7), we determine $d_{max}$. For example, with an input power of 10 dBm and a speaker distance of 1 m from both antennas, we obtained an SNR of 18 dB. Assuming the minimal detection signal-to-noise ratio, $SNR(x=d_{max})$, is zero, this results in a maximum detection distance of approximately 2.8 m, beyond which the signal cannot be detected. For detection at greater distances, we can increase the transmitted power or decrease the antenna beamwidth, thereby increasing the power density of the RF beam.

### 4.2. Respiration Rate Experiment

**Figure 7:**
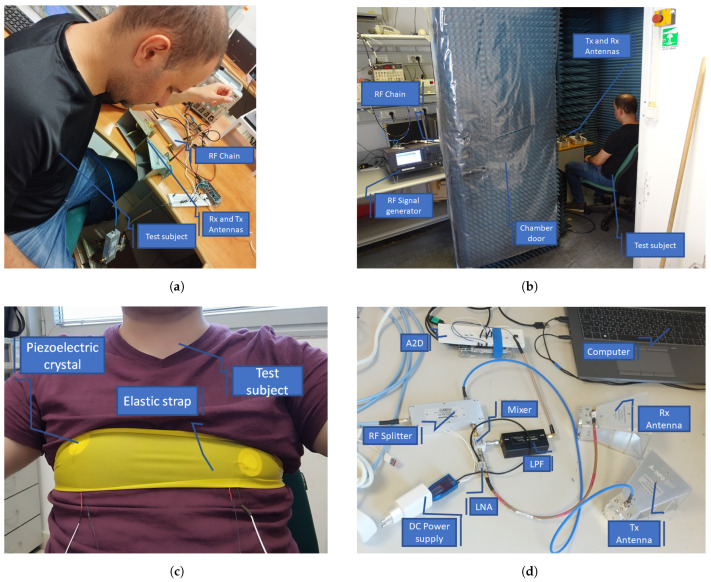
The respiration experiment demonstrates the positioning of antennas towards the subject’s chest (**a**). The same experiment was conducted in both an anechoic chamber (**b**) and an uncontrolled environment (**a**) to demonstrate the ability to detect signals in a noisy environment. The anechoic chamber isolates our measurements from unknown exterior electromagnetic signals due to the radiation-absorbent material coated on its walls. For the validation of remote measurements of the respiration rate, a piezoelectric crystal was placed on the subject’s chest, as shown in (**c**), serving as an additional analog measurement for our system. The complete connections of our system are depicted in (**d**).

The following section describes the respiration rate experiment methodology. A series of tests were conducted on a subject seated in an immobile state on a chair, as shown in Figure 7, in order to eliminate the influence of other body movements. The subject was positioned facing an array of antennas. The data were collected from two different locations using two receiving antennas and showed negligible differences, indicating an ability to capture signals in all directions the antenna is facing. The experiment was performed in a regular office full of modern telecommunication electronics, as shown in Figure 7a, in order to demonstrate the ability of our system to function correctly in an uncontrolled environment and also in an anechoic chamber, as shown in Figure 7b, to show that the signal originated from our system and not from an unknown electronic instrument.

In order to validate the remote measurements of the respiration rate, we positioned a piezoelectric crystal on the subject’s chest, as shown in Figure 7c. This acts as a supplementary analog measurement for our system. Figure 7d provides a detailed depiction of our system’s comprehensive connections, extending from RF connectorized components to A2D and, finally, to the computer, where subsequent analysis took place.

These tests were carried out at three distinct rates of respiration: slow, regular, and hyperventilation. The results were examined by studying the scalogram, which breaks down the frequency components and displays their behavior over time. The respiration rate was verified using a commercial breathing device for athletes, which was strapped to the subject’s torso and was found to be similar to the measured signal with perfect precision.

**Figure 8:**
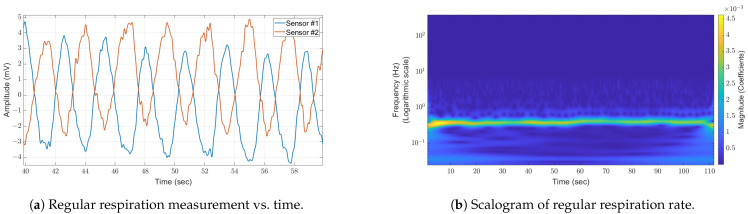
The process of respiration over a period of time (**a**), as recorded by two antennas receiving signals during normal breathing patterns. The scalogram of regular respiration (**b**), captured from one of the antennas, reveals a clear signal variation in the low-frequency region. This fluctuation is attributed to the varying respiration rates over time.

Following the initial rough filtering of the analog data via a low-pass filter, the signal is depicted in Figure 8a. Given that the two receiving sensors are situated at different locations, they capture distinct signals characterized by varying Doppler frequencies and amplitudes.

The scalogram of a regular respiration rate is shown in Figure 8b and shows a relatively constant respiration rate at a lower frequency; the logarithmic scale of the frequency axis enables the separation of the low-frequency components and, therefore, is suitable for the analysis of nonstationary signals from the human body.

The summarized results of the respiration experiment for three different respiratory rates are presented in Table 2. These results have been validated. The frequencies observed are derived from the scalogram, and the breaths per minute were manually collected by observing the subject and counting the number of breaths per minute by recording the data.

**Figure 9:**
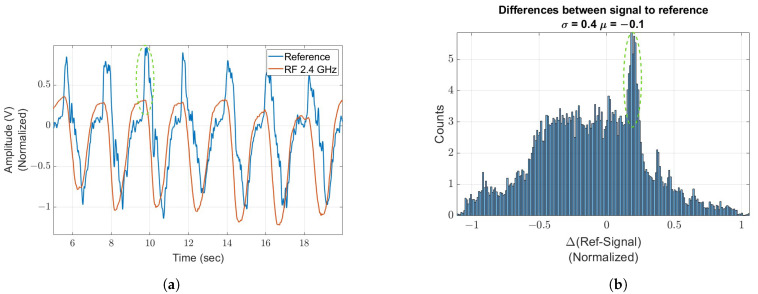
Respiratory signal captured using radio frequency with the reference signal derived from a piezoelectric sensor attached to the subject’s torso (**a**), accompanied by a histogram that illustrates the variances between the two signals (**b**). The green circles indicate a point of the piezoelectric sensor’s high dependency on torso placement, where increased pressure results in a higher magnitude. (**a**) Radio frequency signal alongside the reference signal. (**b**) Histogram of the discrepancies between the signal and the reference.

Breathing detection was verified using an alternative measurement that relies on stress points on the torso, as described in Figure 9, utilizing a piezoelectric sensor. The sensor’s performance is significantly influenced by its placement on the torso, given the non-uniform nature of the breathing activity across the torso. A minor discrepancy, highlighted by a green circle in Figure 9a, is observed and marked. The histogram presented in Figure 9b illustrates the slight difference in the accuracy measure between both methods (in association with the statistical data), exhibiting a standard deviation of 0.4, which describes the spread of the data, and a median of −0.1, which represents the almost constant bias between both measurements. This confirms that the signal measured, indeed, corresponds with breathing activity.

### 4.3. Heart Rate Experiment

**Figure 10:**
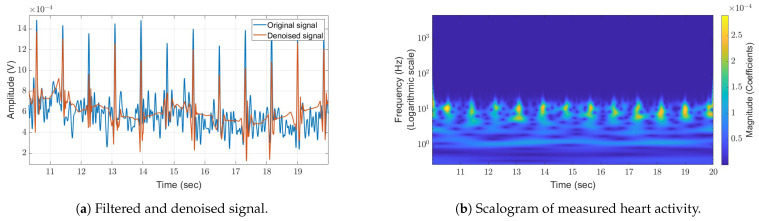
Measured heart rate detection using wavelet denoising (**a**) involves the display of the original signal after it has undergone coarse filtering. The scalogram of measured data (**b**) displays a constant heart rate of 60 bpm over time. These data are presented after the high-frequency background noise has been filtered out during the preprocessing stage.

The following section describes the methodology of the heart rate measuring experiment. In this experiment, the subject was required to remain motionless on a chair and maintain a held breath to isolate the heart rate from respiratory movements. The antennas were strategically positioned toward the subject’s chest area at a distance, *d*, of about 1 m in the far field of the antenna to monitor cardiac activity. It was anticipated that the pulses received would correspond to the rate of a resting human at about 60 beats per minute. The raw data, presented as a scalogram without the application of wavelet denoising, can be seen in Figure 10b. Given the small magnitude of the signal in this scenario, minor body movements can obscure it. Therefore, the use of band-pass filtering (BPF), supplemented with wavelet denoising and peak detection with a defined threshold, is essential for detecting heart activity. This method was employed, and the results are illustrated in Figure 10a, where a heart rate of about 60 beats per minute can be observed. The results were verified using a commercial sports wristwatch that records heart activity. The results were found to be accurate, indicating the ability of our system to capture heart rate with good reliability.

### 4.4. Antenna Frequency Response Experiment

**Figure 11:**
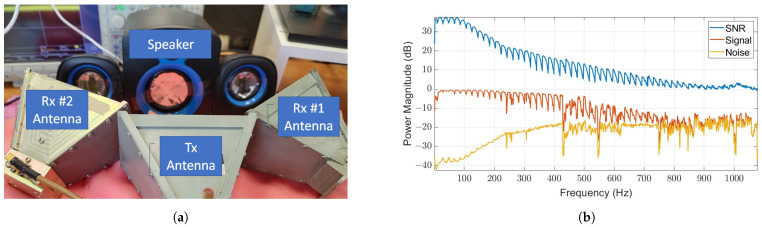
RF signals are transmitted and then reflected off the surface of the speaker’s membrane (**a**). These reflected signals are then detected by two differently positioned receiving antennas for the purpose of spatial representation. The antenna frequency response (**b**) of the speaker membrane exhibits an acceptable SNR of above zero for frequencies up to 850 Hz. Beyond this point, the SNR starts to decrease below zero, eventually reaching the noise level where the signal becomes indistinguishable. The variations in the reflected signal stem from its inherent response when it generates a specific frequency. A perfect speaker, on the other hand, would emit such a signal without these variations.

In order to assess the precision of our system’s Doppler frequency detection and evaluate its existing capabilities, we carried out an experiment involving a speaker membrane, as shown in Figure 11a. In this experiment, a signal, y(t), characterized by a specific frequency, *f*, as depicted in Equation (8), was used to modulate the speaker. Every frequency modulation was captured for a minimum duration of 1 min, and a signal-to-background noise ratio (SNR) was computed. This SNR was then utilized to build the frequency response depicted in Figure 11b. This was carried out for all frequencies until the point where the SNR declined, indicating that our system is currently capable of detecting frequencies below 850 Hz. The hypothesis suggests that narrowing the beamwidth or amplifying the transmission power could potentially enhance the SNR due to the signal’s concentration within a more restricted beam covering the target area with increased power density. For vital bio signs monitoring, this performance is sufficient since most signals of bio signs are in the range of up to 3 Hz. The precision of our system’s Doppler frequency detection is indeed impressive, with an estimated accuracy of ±0.01 Hz. The stability of the signal we emit, derived from our highly stable RF source (Keysight model E8257D) and the in-phase inputs of the RF mixer, contribute significantly to this accuracy. As we utilize CW without any modulation and a stable signal source, we do not expect any signal deviation. The quality of the signal measurement and the minimal noise at low frequencies, as shown in the system frequency response in Figure 11b, further enhance our system’s precision. The assumed accuracy was validated in an experiment where we synthesized a signal using a speaker, as depicted in Figure 11a, with a frequency interval of 0.01 Hz. We were able to identify it using contentious wavelet transformation, which exhibits high accuracy in low frequencies with great precision.

(8) y(t)=A·sin(2πft),

The layout of the experiment is depicted in Figure 11a, where one antenna is responsible for transmission, and two others, equipped with independent A2D circuits, are set up for reception. Despite their different positions relative to the speaker, both antennas should exhibit similar responses. The speaker located in the middle is a subwoofer, which is utilized for the modulation of low frequencies.

### 4.5. Finding the Optimal Transmission Power for Vital Signs Detection

**Figure 12:**
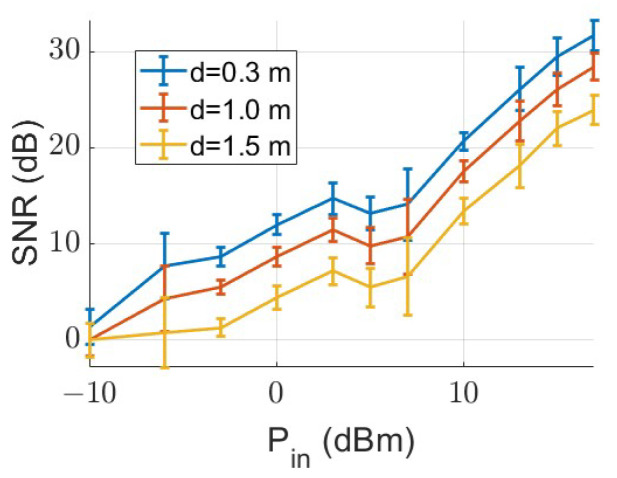
Variation in SNR with source power level for various distances between the antenna source and test subject.

Typical vital signs fluctuate at low frequencies, specifically below 3 Hz. The objective of this experiment was to identify the optimal signal source power parameter that would allow for the detection of these vital signs at a distance denoted as *d*. The experiment was carried out using a speaker that emitted a single tone ranging from 1 to 3 Hz. We adjusted both the signal power and the distance between the antenna and the speaker during the experiment. Data were collected for the median and standard deviation values of the SNR, and a decrease in SNR was observed with increasing distance. Our goal was to utilize the minimum necessary power that still allows for the detection of these signals with a relatively high SNR. Figure 12 presents the results of the experiment. It clearly indicates that we could conveniently utilize a power level of −3 dBm for a target situated within a range of 0.3 to 1.5 m.

### 4.6. Capability of Detection in All Directions

**Figure 13:**
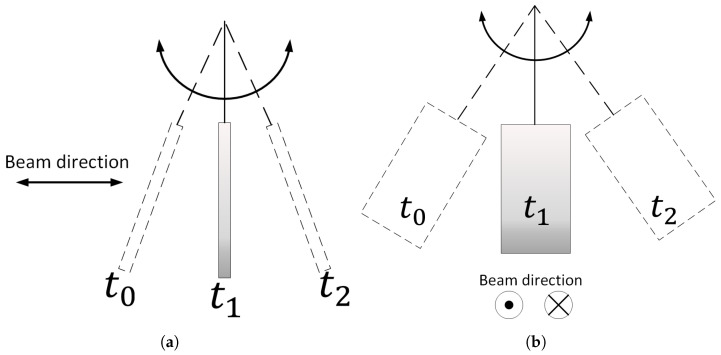
Depiction of pendulum motion over time, where the movement is perpendicular (**a**) and parallel (**b**) to the plane of the antenna. The terms $t_{0}$ to $t_{2}$ describe the temporal position of the pendulum over time where $t_{0}$ is the initial time and $t_{2}$ corresponds to a later time.

In this section, we aim to showcase our system’s capability to detect motion in all directions. This is a significant advancement over conventional radar systems, which can only detect movements perpendicular to the antenna plane, as depicted in Figure 13a. Our system, however, can also detect parallel movements, as illustrated in Figure 13b. In order to validate this capability, we conducted an experiment using a pendulum with a simple square metallic reflective surface moving in two distinct directions.

**Figure 14:**
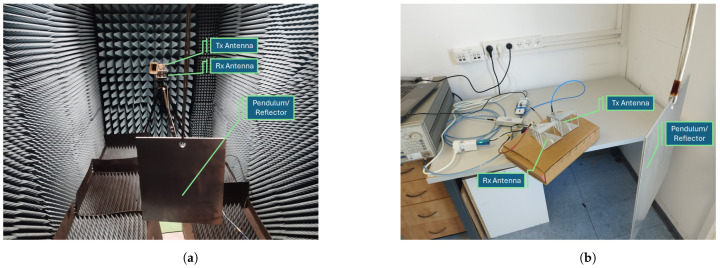
The experimental setup is depicted in two different environments. (**a**) Controlled environment within an anechoic chamber designed to eliminate any interference. (**b**) Uncontrolled environment, specifically a typical office space with surrounding modern electronics.

Our experiment was carried out in two distinct environments: a controlled environment and an uncontrolled environment. The controlled environment, depicted in Figure 14a, was an anechoic chamber designed to eliminate any external interference; in this case, both antennas were mounted on a tripod. On the other hand, the uncontrolled environment, as shown in Figure 14b, was a typical office setting with various modern electronic devices in close proximity, where both antennas were placed on top of a cardboard box. Interestingly, the performance of our experiment remained consistent in both environments, suggesting that any potential external interference did not impact our results.

**Figure 15:**
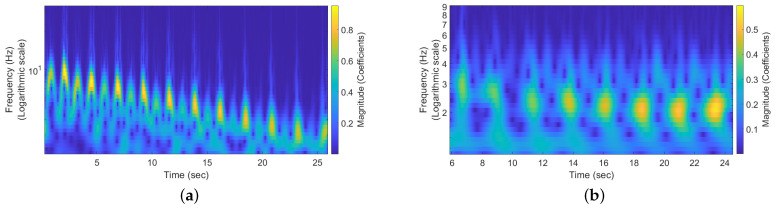
The scalogram showcases the captured signals from a pendulum moving in two separate directions: perpendicular (**a**) and parallel (**b**) to the antenna’s plane. This representation allows us to observe a damping effect in the pendulum’s motion over time, as the frequency components exhibit a decrease, indicating a slowdown in the pendulum’s movement. (**a**) The scalogram illustrates the motion of a pendulum, which moves perpendicular to the plane of the antenna. (**b**) The scalogram depicts the pendulum’s motion, which occurs alongside the antenna’s plane.

The pendulum’s movements can be discerned by examining the frequency components in Figure 15. The frequency components captured during the perpendicular movement are displayed in Figure 15a, which represents the total movements captured by the beam, from the string holding the reflective surface to the surface itself. Similarly, the parallel movement generates frequency components, as depicted in Figure 15b. These frequency components are lower than those in the perpendicular motion. It is also observable that the perpendicular movement is slightly more pronounced than the parallel movement. A common characteristic of both movements is the damping effect, which is evidenced by the decrease in frequency components over time, indicating a slowdown in the pendulum’s motion. It is also noticeable that in between the peaks of the scalograms, there is a blank spot, indicating the pendulum is reaching its peak position. In both scenarios, the reflective surface remains consistently in front of the antenna, remaining within the beam’s coverage.

### 4.7. Evaluating Breathing Detection across Multiple Orientations

**Figure 16:**
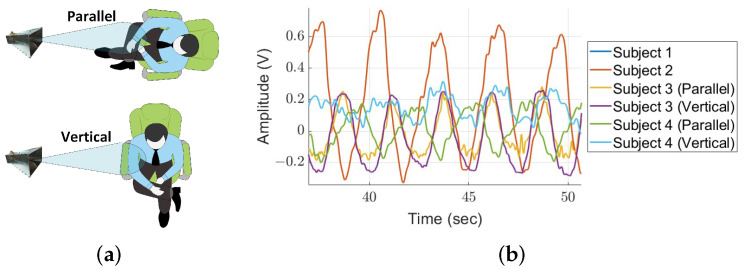
Illustration of the experimental setup: (**a**) participants in parallel and vertical orientations relative to the antenna. The breathing patterns of the participants, displayed as amplitude versus time (**b**), highlighting the differences in chest movements among the subjects.

The purpose of this subsection is to investigate the measurement of different individuals and their behavior, along with the detection performance in both directions, as illustrated in Figure 16a. In this figure, the subject is either facing the antenna (parallel orientation) or positioned at a 90-degree angle to the antenna (vertical orientation). In this experiment, four participants were instructed to sit immobilized in a chair and breathe regularly. The first two participants faced the antenna in parallel, while the third and fourth participants were measured simultaneously in both parallel and vertical orientations using an additional set of antennas. The nature of each participant’s breathing, shown as amplitude versus time in Figure 16b, was found to be different. Subject number 2 exhibited larger chest movements, whereas subject number 4 breathed lightly. This demonstrates that we can measure breathing in all directions and detect different test subjects.

## 5. Discussion and Conclusions

In conclusion, our research emphasizes the innovative capability of our new measurement technique, which stands out from conventional radar systems that are restricted by directional sensitivity. This technique precisely detects micro-movements in all directions, marking a significant advancement in the noncontact evaluation of vital bio signs.

Our experimental findings demonstrate the effectiveness of the proposed method in accurately measuring physiological parameters such as respiration and heart rate. Specifically, we conducted a series of experiments that validated our approach, showing consistent results across various scenarios. For instance, the data collected during these experiments indicated a strong correlation between the measured signals and the expected physiological responses, confirming the reliability of our technique.

Additionally, the simulations conducted using the CWT method provided valuable insights into the behavior of the signals over time. The CWT allowed us to analyze nonstationary signals effectively, revealing the temporal dynamics of the physiological indicators. The scalograms generated from the CWT analysis illustrated the frequency components of the signals, demonstrating how they varied with time and providing a nuanced understanding of respiratory and cardiac activity.

Despite these positive findings, we acknowledge that further research is essential to continually improve and refine the proposed methodology. This may involve optimizing the measurement techniques, exploring additional parameters, and enhancing the application of microwave radiation for more comprehensive and accurate vital bio signs measurements. Moreover, in future work, we plan to utilize a laser as an additional sensor that can provide us with more information on the subject’s condition. We understand that ongoing research will contribute to its continued development and efficacy.

In summary, the combination of our experimental results and the insights gained from the CWT simulations supports the feasibility and effectiveness of using 2.4 GHz microwave radiation for measuring vital bio signs through human tissues. These findings lay a robust foundation for future research and applications in the field of human physiology.

## Figures and Tables

**Table 1:** Complex dielectric constants and conductivity of 23 human tissues located in the abdominal and upper-chest regions at a microwave frequency of 2.4 GHz. The data for these microwave frequencies were collected from the literature [33] and fitted using a multi-pole Debye model.

Tissue	Relative Dielectric Constant	Relative Loss Factor	Conductivity
	$K^{\prime }$	$K^{\prime \prime }$	$\sigma \frac{S}{m}$
Aorta	42.659	10.612	1.417
Bladder	18.05	5.057	0.675
Blood	58.524	18.748	2.503
Blood vessel	42.679	10.607	1.416
Bone cancellous	18.603	5.876	0.785
Bone cortical	11.428	2.798	0.374
Bone marrow	5.327	0.704	0.094
Breast fat	5.182	0.975	0.13
Fat	5.293	0.758	0.101
Heart	55.023	16.583	2.214
Kidney	53.078	17.583	2.396
Liver	43.197	12.541	1.674
Lung deflated	48.339	12.345	1.648
Lung inflated	20.507	5.976	0.798
Muscle	52.995	12.831	1.713
Ovary	45.101	16.801	2.243
Pancreas	57.307	14.437	1.928
Skin dry	37.961	10.857	1.45
Small intestine	54.626	23.272	3.107
Spinal cord	30.204	8.024	1.071
Spleen	52.546	16.511	2.205
Stomach	62.403	16.348	2.183
Tendon	43.402	12.354	1.649

**Table 2:** The summary of the respiration experiment test results indicates a strong correlation between the measured respiration rate and the actual frequency.

Respiratory Rate	Measured (Breath per Minute)	Frequency Range (Hz)
Slow	10	0.16±0.01
Regular	17	0.28±0.01
Hyperventilation	26	0.43±0.01

## Data Availability

The data that support the findings of this study are included in the article. Further information is available from the corresponding author upon reasonable request.

## Abbreviations

The following abbreviations are used in this manuscript:

A2D	Analog to digital
BPF	Band-pass filtering
bpm	Beats per minute
CNC	Cellulose nanocrystals
CSI	Channel state information
CW	Continuous wave
CWT	Continuous wavelet transfer
dB	Decibels
ECG	Electrocardiogram
FMCW	Frequency-modulated continuous wave
FSPL	Free-space path loss
IQ	In-phase and quadrature
LNA	Low-noise amplifier
LPF	Low-pass filter
PENGs	Piezoelectric nanogenerators
PWV	Pulse wave velocity
Py-PNG	Pyro-piezoelectric nanogenerator
RF	Radio frequency
RX	Receiver
SNR	Signal-to-noise ratio
SSA	Singular spectrum analysis
STFT	Short-time Fourier transform
Tx	Transmitter
UWB	Ultra-wideband
VMD	Variational mode decomposition
VSWR	Voltage standing wave ratio
VT	Ventricular tachycardia
WiFi	Wireless fidelity
